# Impact of KRAS^G12D^ subtype and concurrent pathogenic mutations on advanced non-small cell lung cancer outcomes

**DOI:** 10.1007/s12094-023-03279-2

**Published:** 2023-07-25

**Authors:** Enrique Caballé-Perez, Norma Hernández-Pedro, Maritza Ramos-Ramírez, Pedro Barrios-Bernal, Eunice Romero-Núñez, José Lucio-Lozada, Santiago Ávila-Ríos, Gustavo Reyes-Terán, Andrés F. Cardona, Oscar Arrieta

**Affiliations:** 1 Thoracic Oncology Unit, Instituto Nacional de Cancerología (INCan), Mexico City, Mexico; 2 Personalized Medicine Laboratory, Instituto Nacional de Cancerología (INCan), Mexico City, Mexico; 3 Instituto Nacional de Enfermedades Respiratorias, Mexico City, Mexico; 4 Thoracic Oncology Unit and Direction of Research, Science and Education, Luis Carlos Sarmiento Angulo, Cancer Treatment and Research Center (CTIC), Bogotá, Colombia; 5 Clinical and Translational Oncology Group, Clínica del Country, Bogotá, Colombia; 6 Molecular Oncology and Biology Systems Research Group (Fox-G), Universidad El Bosque, Bogotá, Colombia

**Keywords:** KRAS G12D, Non-small cell lung carcinoma, Comutations, TP53, STK11, Immunotherapy

## Abstract

**Purpose:**

Mutations in the Kirsten rat sarcoma viral (KRAS) oncogene constitute a significant driver of lung adenocarcinoma, present in 10–40% of patients, which exhibit heterogeneous clinical outcomes, mainly driven by concurrent genetic alterations. However, characterization of KRAS mutational subtypes and their impact on clinical outcomes in Latin America is limited.

**Methods:**

A cohort study was conducted at the National Cancer Institute (INCan) of Mexico. Individuals with advance-staged of adenocarcinoma and KRAS mutations, detected by next-generation sequencing, having undergone at least one line of therapy were included for analysis. Clinical and pathological characteristics were retrieved from institutional database from June 2014 to March 2023.

**Results:**

KRAS was identified in fifty-four (15.6%) of 346 patients, among which 50 cases were included for analysis. KRAS^G12D^ (n = 16, 32%) and KRAS^G12C^ (n = 16, 32%) represented the most prevalent subtypes. KRAS^G12D^ mutations were associated with female (*p* = 0.018), never smokers (*p* = 0.108), and concurrences with EGFR (25.0% vs. 17.6%, *p* = 0.124) and CDKN2A (18.8% vs. 14.7%, *p* = 0.157). KRAS^G12D^ patients showed a better ORR (66.6% vs. 30.0%; OR 4.66, 95% CI 1.23–17.60, *p* = 0.023) and on multivariate analysis was significantly associated with better PFS (HR 0.36, 95% CI 0.16–0.80; *p* = 0.012) and OS (HR 0.24, 95% CI 0.08–0.70; *p* = 0.009).

**Conclusions:**

To our knowledge, this study represents the first effort to comprehensively characterize the molecular heterogeneity of KRAS-mutant NSCLC in Latin American patients. Our data reinforce the current view that KRAS-mutated NSCLC is not a single oncogene-driven disease and emphasizes the prognostic impact of diverse molecular profiles in this genomically defined subset of NSCLC. Further validation is warranted in larger multicenter Latin American cohorts to confirm our findings.

**Supplementary Information:**

The online version contains supplementary material available at 10.1007/s12094-023-03279-2.

## Introduction

Lung cancer (LC) is the leading cause of cancer-related mortality worldwide, with 1.70 million deaths and 2.2 million new cases in 2020 [1]. In recent years, mutational characterization of lung cancer has improved its therapeutic outcomes. Mutations in Kirsten rat sarcoma viral oncogene homolog (KRAS) represent the most frequent oncogene alterations in NSCLC, with variable incidences across ethnicities, being less prevalently in East Asian (5–11%) and Latin American countries (14%) than in Caucasian patients (25–40%) [2, 3]. Most KRAS alterations occur in codon 12 (80%), mainly as a substitution of glycine by cysteine (G12C) in 39–40% of cases, followed by valine (G12V) in 17–21%, aspartate (G12D) in 14–17%, or alanine (G12A) in 9–10% [4]. These mutations impair GTP hydrolysis by GTPase-activating proteins (GAPs), triggering KRAS-derived signaling through MAPK and PI3K-AKT-mTOR pathways. Despite their prevalence, the prognostic impact of KRAS mutations remains uncertain owing to their highly heterogeneous clinical course and variable response to current therapies. For instance, KRAS^G12D^ mutation has been linked to inferior clinical outcomes among patients with KRAS-mutated NSCLC who underwent PD-L1 blockade [5]. Coexisting genomic alterations may explain this prognostic significance, potentially representing predictive biomarkers in immunotherapy setting. These include mutations in tumor protein 53 (TP53), serine/threonine 11 (STK11), and Kelch-like ECH-associated protein 1 (KEAP1), alterations in Mesenchymal Epithelial Transition (MET), and loss of cyclin-dependent kinase 2A (CDKN2A) [6]. Understanding the role of co-occurring genomic alterations in KRAS-mutated tumors is critical for developing effective personalized treatments and improving patient’s outcomes; however, they have shown inconsistent effects across various studies [7]. Therefore, this study aims to analyze clinicopathological and genomic characteristics of Latin American patients with KRAS-mutated advanced NSCLC, focusing on their impact on therapeutic outcomes.

## Patients and methods

An observational longitudinal cohort study was conducted on 346 patients previously diagnosed with advanced NSCLC from June 2014 to March 2023 at the Thoracic Oncology Unit of the Instituto Nacional de Cancerología (INCan). Consecutive patients with confirmed advanced NSCLC harboring a KRAS mutation detected by next-generation sequencing were eligible. Patients who received at least one line of anticancer therapy were included in the analysis. Response was evaluated according to RECIST v1.1 [8]. Clinical and pathological data, including baseline patient characteristics, treatment regimens, therapeutic efficacies, and survival, were collected from electronic medical records. This study protocol was approved by the institutional review board (CEI/1375/19).

### Samples processing

Available formalin-fixed and paraffin-embedded tissues (FFPE) were analyzed by the institutional pathology department, which performed histologic diagnosis and quantification of the percentage of neoplastic cellularity in each sample. The procedure for DNA extraction and purification was carried out using QI Amp DNA FFPE tissue kit (QIAGEN, Netherlands, USA, Cat. Number: 56404). Concentration and integrity of genetic material were measured using a 2100 bioanalyzer system (Agilent, California, EUA, #G2939BA). Three different kits were used to evaluate KRAS mutations and their concurrences: AmpliSeq Cancer HotSpot Panel v2, TruSeq Amplicon Cancer Panel, and Foundation One (FO). Gene mutations analysis included those with nonsense mutations, frameshift, and in-frame insertion-deletion mutations (indels), splice site mutations, and missense mutations defined as oncogenic in cBio Cancer Genomics Portal repository [9].

### Next-generation sequencing methodology

The TruSeq Amplicon Cancer Panel (Illumina, California, EUA, #FC-130-1008) was used to constitute the genetic library for 48 cancer-related genes. Also, there was used AmpliSeq Cancer HotSpot Panel v2 (Illumina, California, EUA, #20019161), which contained 50 genes associated with cancer, and externally, 175 samples were analyzed by FO panel (Roche, Basilea, Suiza, PLA code: 0037U), which detects abnormalities in 236 genes, and 19 rearrangements. Additionally, quality control of concentration and size of genomic libraries was performed using the Quantus fluorometer (Promega, Wisconsin, EUA, #E6150), as well as a 2100 Bioanalyzer system (Agilent, California, EUA, #G2939BA). Then, targeted sequencing was performed in a MiSeq instrument (Illumina, California, EUA, #SY-410-1003), with an average sequencing depth per base of 1000X.

### Statistical analyses

Continuous variables were reported as means and standard deviations (SD), or medians and interquartile ranges (IQR) based on data distribution assessed by Kolmogorov–Smirnov Test. According to data distribution, comparisons for continuous variables between groups were evaluated using the Student’s t-test or Mann–Whitney U-test. Categorical variables were reported as frequencies and proportions, and comparisons among them were analyzed by χ2 test or Fisher exact test. Conditional odds ratios (OR) and Fisher’s exact test* p*-values were used to assess co-occurrence and mutual exclusivity for genes among KRAS mutated and wild-type cases. Clinical and genomic characteristics associated with ORR were presented as OR estimated using logistic regression models. Kaplan Meier curves were used to evaluate median PFS and OS. The log rank test and Cox's proportional hazards model was used to test differences over time. All *p*-values were two-sided, with statistical significance defined as *p* < 0.05. All statistical analyses were conducted using Stata/MP 14.0 for Mac (StataCorp LP, 2015), and GraphPad Prism 9.0.1 for macOS (GraphPad Software, 2021) was used for plotting.

## Results

### Baseline characteristics of NSCLC cohort with KRAS mutations

**Fig. 1:**
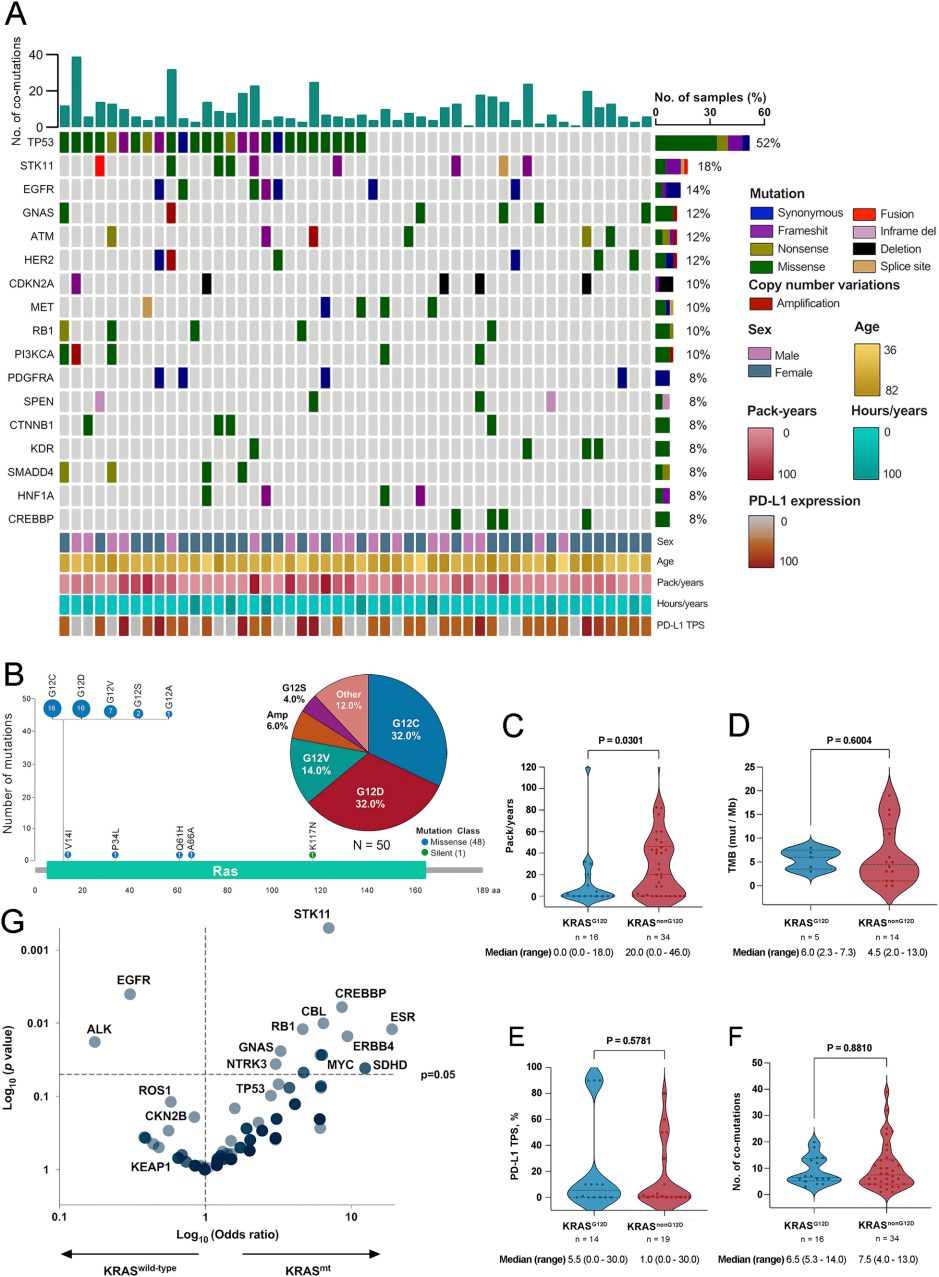
**A**, clinical characteristics and comutations of KRAS-mutated NSCLC patients**. B**, structural representation, and frequency of KRAS mutations. **C**, smoking history according to packs per year in KRAS^G12D^ or KRAS^non-G12D^ groups. **D**, Tumor mutational burden in KRAS^G12D^ and KRAS^non-G12D^ groups. **E**, assessment of PD-L1 TPS expression according to KRAS^G12D^ mutation. **F**, number of commutations between individuals with in KRAS^G12D^ and KRAS^non-G12D^. mutations **G**, Association probability of KRAS with other driver genes. KRAS, Kirsten rat sarcoma viral oncogene homolog. G12C, missense substitution of glycine for cysteine. G12D, missense substitution of glycine for aspartate. G12V, missense substitution of glycine for valine. G12A, missense substitution of glycine for alanine. G12S, missense substitution of glycine for serine. V14I, missense substitution of valine for isoleucine. P34L, missense substitution of proline for leucine. Q61H, missense substitution of glutamine for histidine, K117N, missense substitution of lysine for asparagine. A66A, silent mutation coding for alanine in both original and mutated forms. Amp, amplification. TP53, tumor protein p53. STK11, Serine/Threonine Kinase 11. EGFR, epidermal growth factor receptor gene. EGFR mutations detected were: G719S and S768l (n = 1), Q787Q (n = 5), G288Vfs*5 (n = 1) and G403E (n = 1). GNAS, guanine nucleotide-binding protein, alpha stimulating complex locus. ATM, ataxia telangiectasia mutated. HER2, human epidermal growth factor receptor 2. CDKN2A, Cyclin-Dependent Kinase Inhibitor 2A. MET, mesenchymal-epithelial transition factor. RB1, Retinoblastoma 1. PI3KCA, phosphatidylinositol-4,5-bisphosphate 3-kinase catalytic subunit alpha. PDGFRA, platelet-derived growth factor receptor alpha. SPEN, Spen Family Transcriptional Repressor. CTNNB1, Catenin Beta 1. KDR, Kinase Insert Domain Receptor. SMADD4, SMA- and MAD-related protein 4. BRCA1, breast cancer gene. HNF1A, hepatocyte nuclear factor 1 alpha. CREBBP, CREB Binding Protein. ESR, estrogen receptor 1. ERBBB4, Erb-B2 Receptor Tyrosine Kinase 4. MYC, MYC Proto-Oncogene. SDHD, Succinate Dehydrogenase Complex Subunit **D**. CBL, Casitas B-lineage Lymphoma. NTRK3, neurotrophic receptor tyrosine kinase 3. CDKN2B, cyclin dependent kinase inhibitor 2B. ROS1, ROS Proto-Oncogene 1. KEAP1, Kelch-like ECH-associated protein 1. ALK, anaplastic lymphoma kinase. TMB, tumor mutational burden. PD-L1, programmed death ligand 1. TPS, tumor proportion score. Tobacco exposure index was calculated by multiplying smoked cigarette packs and years of exposure, then this result was divided into 20. Comparisons in figures C-F were performed using Mann–Whitney test according to normal distribution determined by the Kolmogorov–Smirnov test. Significant *p* values were defined as less than 0.05

Among 346 patients with advanced NSCLC 15.6% (n = 54) harboring KRAS mutations were identified, and 50 cases were included in the analysis (Supplementary Figure S1). Main clinical, demographic, histological, and molecular characteristics of the entire cohort are summarized in Fig. 1A. Overall, mean age was 62.8 years (SD ± 11.6), 64.0% (n = 32) were female, 60% (n = 30) were current or former smokers with a median pack-year of 9.6 (range 0.0–43.0), and 78% (n = 39) had an ECOG performance status (PS) of 0–1 (Table 1). The most common KRAS mutations subtypes were G12D and G12C (n = 16, 32.0%, respectively), followed by G12V (n = 7, 14.0%) (Fig. 1B). Baseline clinicopathological characteristics of patients stratified by KRAS^G12D^ or KRAS^non-G12D^ status are shown in Table 1. Clinical factors significantly associated with the KRAS^G12D^ were female sex (87.5% vs. 52.9%, *p* = 0.018) and contralateral lung metastases (62.5% vs. 29.4%, *p* = 0.026) (Table 1). Characteristics of patients with KRAS^G12C^ or KRAS^non-G12C^ subtypes are summarized in Supplementary Table S1.

**Table 1:** Baseline characteristics, stratified according to KRAS^G12D^ and KRAS^non-G12D^

		Total N = 50 (100.0)	KRAS^G12D^ n = 16 (32.0)	KRAS^non−G12D^ n = 34 (68.0)	*p*-value
Age, mean (SD)		62.8 (11.6)	60.9 (12.8)	63.7 (11.1)	0.213^a^
Sex, n (%)	Male	18 (36.0)	2 (12.5)	16 (47.1)	
	Female	32 (64.0)	14 (87.5)	18 (52.9)	**0.018**^**a**^
ECOG PS, n (%)	0–1	39 (78.0)	11 (68.7)	28 (82.3)	
	≥2	11 (22.0)	5 (31.3)	6 (17.7)	0.279^c^
Smoking status, n (%)	Current/former	30 (60.0)	7 (43.7)	23 (67.7)	
	Never	20 (40.0)	9 (56.3)	11 (32.3)	0.108^c^
Pack-years, median (range)		9.6 (0.0 – 43.0)	0.0 (0.0 – 18.0)	20.0 (0.0 – 46.0)	**0.030**^**b**^
WSE, n (%)	Positive	13 (26.0)	5 (31.2)	8 (23.5)	
	Negative	37 (74.0)	11 (68.8)	26 (76.5)	0.562^a^
Hours/years, median (range)		0.0 (0.0 – 4.37)	0.0 (0.0 – 18.0)	20.0 (0.0 – 46.0)	0.832^b^
Histology, n (%)	Adenocarcinoma	49 (98.0)	15 (93.8)	34 (100.0)	0.141^c^
Adenocarcinoma classification, n (%) (n = 45)	LEP predominant	10 (22.2)	3 (21.4)	7 (22.6)	
	ACN predominant	13 (28.9)	3 (21.4)	10 (32.2)	
	PAP predominant	4 (8.9)	3 (21.4)	1 (3.23)	
	MCP predominant	1 (2.2)	1 (7.4)	0 (0.0)	
	SOL predominant	17 (37.8)	4 (28.6)	13 (41.9)	0.168^d^
Clinical stage, n (%)	Stage IIIB-C	12 (24.0)	3 (18.8)	9 (26.5)	
	Stage IVA-IVB	38 (76.0)	13 (81.2)	25 (73.5)	0.551^c^
PD-L1 expression, n (%), (n = 33)	TPS < 1%	15 (45.5)	6 (50.0)	9 (47.4)	
	TPS 1%	18 (54.5)	8 (57.1)	10 (52.6)	0.797^c^
PD-L1 expression, n (%), (n = 33)	TPS < 50%	27 (81.8)	12 (85.7)	15 (78.9)	
	TPS ≥ 50%	6 (18.2)	2 (14.3)	4 (21.1)	0.490^d^
PD-L1 TPS, median (range)		1.0 (0.0 – 20.0)	5.5 (0.0 – 30.0)	1.0 (0.0 – 30.0)	0.578^b^
	Not assessed	17	2	15	
TMB, median (range) (n = 19)		5.0 (3.0 – 8.0)	6.0 (2.3 – 7.3)	4.5 (2.0 – 13.0)	0.600^b^
TMB, n (%) (n = 19)	< 10 mts/MB	14 (73.7)	6 (100.0)	8 (61.5)	
	≥ 10 mts/MB	5 (26.3)	0 (0.0)	5 (38.5)	0.077^**a**^
	Not assessed	31	10	21	
No. co-occurring mutations, median (range)		7.0 (4.0 -13.3)	6.5 (5.3 – 14.0)	7.5 (4.0 -13.0)	0.881^b^
No. co-occurring mutations, n (%)	< 5 mts	13 (26.0)	3 (18.8)	10 (29.4)	
	≥5 mts	37 (74.0)	13 (81.2)	24 (70.6)	0.423^a^
Metastatic sites, n (%) (n = 38)	Lymph nodes	10 (20.0)	5 (31.2)	5 (14.7)	0.172^a^
	Contralateral lung	20 (40.0)	10 (62.5)	10 (29.4)	**0.026**^**a**^
	Pleura	7 (14.0)	2 (12.5)	5 (14.7)	0.834^†^
	Bone	16 (32.0)	6 (37.5)	10 (29.4)	0.567^c^
	CNS	8 (16.0)	3 (18.8)	5 (14.7)	0.716^c^
	Liver	5 (10.0)	2 (12.5)	3 (8.8)	0.686^c^
	Adrenal	9 (18.0)	3 (18.8)	6 (17.7)	0.925^c^
Number of metastatic sites, n (%) (n = 38)	1 site	18 (42.9)	8 (53.3)	10 (37.0)	
	2 sites	14 (33.3)	2 (13.3)	12 (44.4)	
	≥ 3 sites	10 (23.8)	5 (33.3)	5 (18.5)	0.117^c^
First-line treatment, n (%) (n = 50)	Anti PD-L1 monotherapy	2 (4.0)	0.0 (0.0)	2 (2.9)	
	Anti PD-L1 monotherapy + platinum basedchemotherapy	11 (22.0)	4 (25.0)	7 (20.6)	
	Platinum-based chemo therapy	36 (72.0)	12 (75.0)	24 (70.6)	
	Targeted therapy	1 (2.0)	0.0 (0.0)	1 (2.94)	0.674^c^
Second-line treatment, n (%) (n = 24)	Present	24 (48.0)	8 (50.0)	16 (47.1)	0.846^c^
	Ant iPD-L1 monotherapy	6 (25.0)	1 (12.5)	5 (31.3)	
	Chemotherapy	16 (66.7)	6 (75.0)	10 (62.5)	
	Targeted therapy	2 (8.3)	1 (12.5)	1 (6.3)	0.659^d^

KRAS, Kirsten rat sarcoma viral oncogene homolog, G12D missense substitution of glycine for aspartate, ECOG eastern cooperative oncology group performance status, WSE wood smoke exposure, LEP lepidic, CAN acinar, PAP papillary, MCP micropapillary, SOL solid, TPS tumor proportion score, PD-L1 TPS programmed death ligand 1 tumor proportion score, TMB tumor mutational burden, EGFR epidermal growth factor receptor, TKI tyrosine kinase inhibitor, CNS central nervous system, Mts mutations, MB megabase. Comparisons were made using: ^a^ t-test or ^b^ Mann–Whitney test according to normal distribution determined by the Kolmogorov–Smirnov test. Nominal variables were analyzed by ^c^ Pearson Chi-Square test, except when small size of sample (n < 5) required using ^d^ Fisher's exact test. Significance was set at *p* < 0.05 (two-sided), and shown as bold values in tables

### Association between KRAS subtype and smoking status, TMB or PD-L1 expression

Among patients with known pack-year smoking data, median pack-years were significantly lower among KRAS^G12D^ patients (0.0 vs. 20.0, *p* = 0.030) (Fig. 1C). In 19 patients harboring KRAS mutations with evaluable TMB, 31.5% (n = 6) had TMB-high (TMB ≥ 10 Muts/Mb). There were no differences regarding median TMB according to KRAS subtype (6.0 vs. 4.5, p = 0.600) (Fig. 1D). Immunohistochemical results of PD-L1 expression by tumor proportion score (TPS) were available for 33 of 66 samples (66.0%). A total of 18 patients (54.5%) were characterized as PD-L1 positive (TPS ≥ 1%), comprising 6 patients (18.2%) with high PD-L1 expression (TPS ≥ 50%). Median PD-L1 expression was similar across KRAS^G12D^ and KRAS^non−G12D^ cases (5.5% vs. 1.0%, p = 0.578) (Fig. 1E). Similar results were observed comparing KRAS^G12C^ and KRAS^non−G12C^ individuals (Supplementary Figure S2A–C).

### Co-occurring genomic alterations in NSCLC with KRAS mutations

**Fig. 2:**
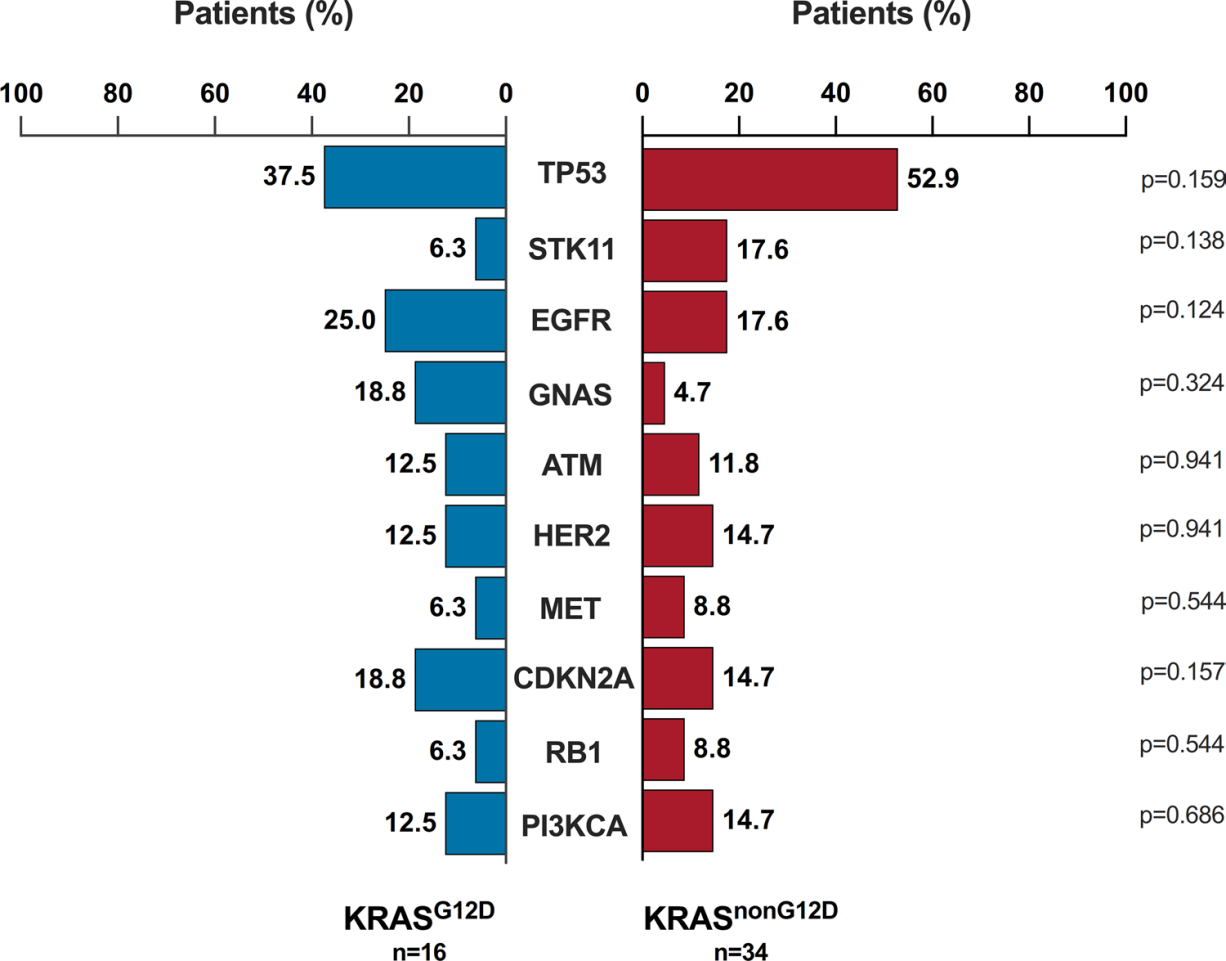
Prevalence of co-mutations in individuals with G12D mutations. KRAS, Kirsten rat sarcoma viral oncogene homolog. G12D, missense substitution of glycine for aspartate. TP53, tumor protein p53. STK11, Serine/Threonine Kinase 11. EGFR, Epidermal Growth Factor Receptor. GNAS, guanine nucleotide-binding protein, alpha stimulating. ATM, Ataxia-Telangiectasia Mutated. HER2, human epidermal growth factor receptor 2. MET, mesenchymal-epithelial transition factor. CDKN2A, Cyclin-Dependent Kinase Inhibitor 2A. RB1, Retinoblastoma 1. PI3KCA, phosphatidylinositol-4,5-bisphosphate 3-kinase catalytic subunit alpha. Comparisons were performed by Pearson Chi-Square test. Significance was set at *p*-values < 0.05

Of the samples analyzed, 96.0% (n = 48) had at least one additional genomic alteration besides KRAS mutation. The most prevalent concurrence identified in the cohort were TP53 (n = 26, 52.0%), STK11 (n = 9, 18%) and EGFR (n = 7, 14%) (Fig. 1A). Compared with wild-type cases, KRAS mutations were significantly associated with comutations in STK11 (OR 7.0, 95% CI 2.48–19.72, *p* < 0.001), RB1 (OR 4.64, 95% CI, 1.41–15.30; *p* = 0.012), GNAS (OR 3.28, 95% CI 1.17–9.22; *p* = 0.024) (Fig. 1G). The median number of co-alterations in tumors harboring KRAS^G12D^ mutation was like that in the KRAS^non−G12D^ subgroup (6.5 vs. 7.5, *p* = 0.881) (Fig. 1F), in contrast, cases with KRAS^G12C^ mutation demonstrated a lower median number of co-alterations compared (Supplementary Figure S2D). Particularly, KRAS^G12D^ cases were enriched in mutations affecting EGFR (25% vs. 17.6%, *p* = 0.124), and deletions of CDKN2A (18.8% vs. 14.7%, *p* = 0.157) genes, whereas loss-of-function mutations in TP53 (52.9% vs. 37.5%, *p* = 0.159) and STK11 (17.6% vs. 6.3%, *p* = 0.138) were more likely to occur in KRAS^non−G12D^ cases (Fig. 2). By contrast, mutations in PI3KCA and alterations in CDKN2A were less likely to occur in KRAS^G12C^ patients compared to KRAS^non−G12C^ cases (Supplementary Figure S3).

### Therapeutic approaches and outcomes in advanced NSCLC with KRAS mutations

Platinum doublet chemotherapy was the most common first-line systemic therapy (n = 36, 72%), followed by chemoimmunotherapy combination (n = 11, 22.0%), anti-PD(L)1 monotherapy (n = 2, 4.0%), and targeted therapy (n = 1, 2.0%). Second-line treatment was administered to 24 (n = 24, 48.0%) patients. A chemotherapy-based regimen was the most common second-line systemic therapy (n = 16, 66.7%), followed by anti-PD(L)1 monotherapy (n = 6, 25.0%), and targeted therapy (n = 2, 8.3%). Treatment regimens are detailed in Supplementary Table S3**.** Patients with KRAS^G12D^ and KRAS^non−G12D^ mutations were similar in terms of first- and second-line treatment modalities (Table 1). At least one line of PD-(L)1 blockade-based therapy was administered in 34% of patients Supplementary Table S3.

**Fig. 3:**
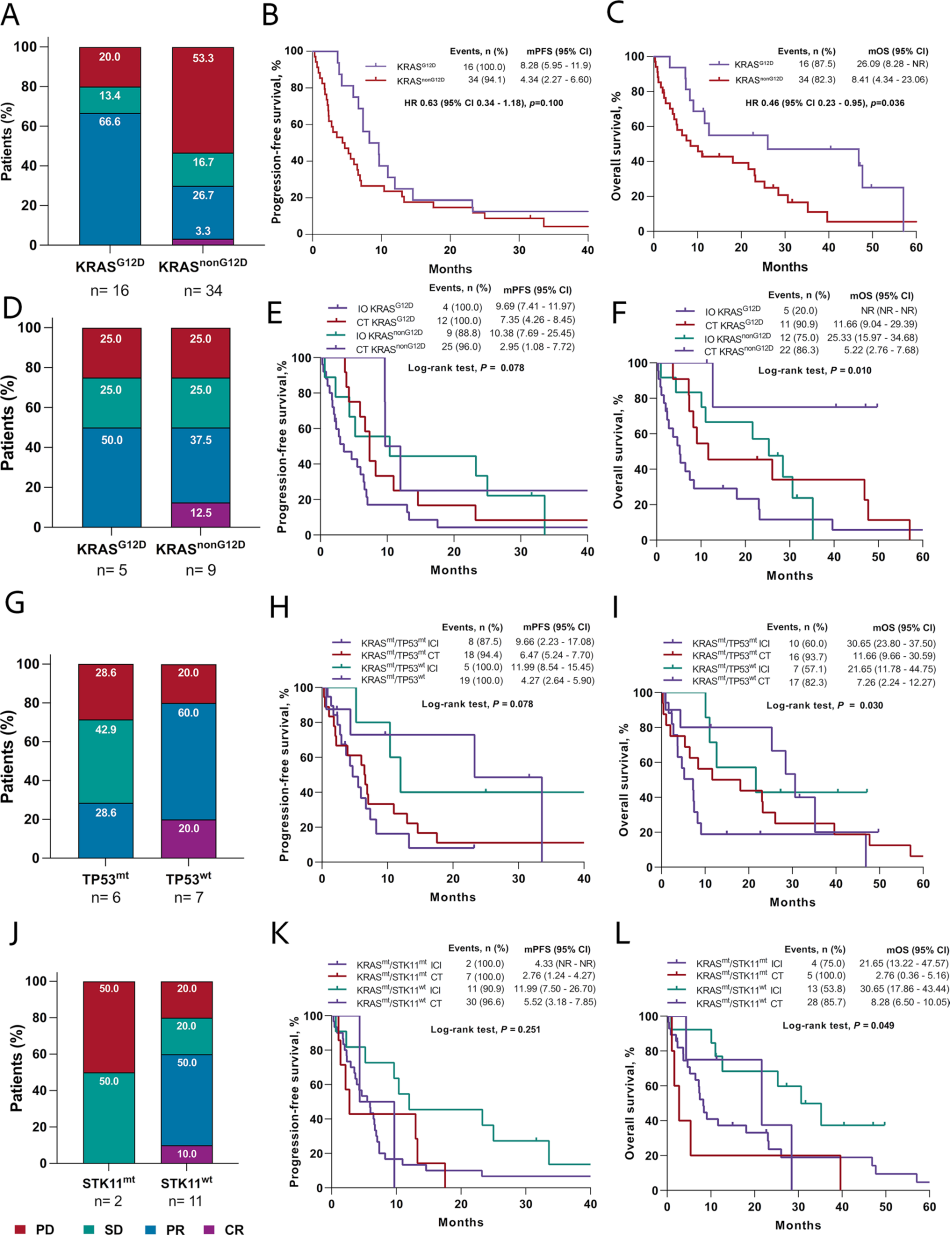
**A**, Type of responses to all treatments according to KRAS^G12D^ mutation. **B**, progression-free survival of individuals with KRAS^G12D^ or KRAS^non−G12D^ mutations after all treatments. **C**, overall survival of patients having KRAS G12D or non-G12D mutations after all treatments. **D**, therapeutic responses to immunotherapy according to KRAS G12D mutation. **E**, progression-free survival of individuals having in KRAS^G12D^ and KRAS^non−G12D^ mutations undergoing immunotherapy or chemotherapy. **F**, overall survival of individuals having in KRAS^G12D^ and KRAS^non−G12D^ mutations undergoing immunotherapy or chemotherapy. **G**, therapeutic responses to immunotherapy in individuals harboring or not TP53 comutation. **H,** progression-free survival of patients having or not comutation with TP53 after immunotherapy or chemotherapy. **I**, overall survival of patients having or not comutation with TP53 after immunotherapy or chemotherapy. **J**, therapeutic responses to immunotherapy of individuals having or not STK11 comutation. **K**, progression-free survival of patients having or not comutation with STK11 after immunotherapy or chemotherapy. **L**, overall survival of patients having or not comutation with STK11 after immunotherapy or chemotherapy. IO, immunotherapy. ICI, immune checkpoint inhibitors. CT, chemotherapy. PFS, progression-free survival. OS, overall survival. KRAS, Kirsten rat sarcoma viral oncogene homolog. G12D, missense substitution of glycine for aspartate. TP53, tumor protein p53. STK11, Serine/Threonine Kinase 11. PFS was calculated from diagnosis to progression to first-line treatment. OS was determined by the period between diagnosis and death for any cause. Log-rank test was performed to determine statistical differences between Kaplan-Meyer curves. *p*<0.05 were considered as significative

Formal response assessments were available for 90.0% (n = 45) of cases; overall, 42.0% (95% CI, 27.7–57.8) patients had confirmed objective responses, of which 2.0% had a complete response; 57.7% of individuals achieved disease control rate (DCR) (95% CI, 42.2–72.3). According to KRAS subtype, there was a greater ORR among KRAS^G12D^ patients (66.6% vs. 30.0%; OR 4.66, 95% CI 1.23–17.60*, p* = 0.023) compared with KRAS^non−G12D^ cases (Fig. 3A). Differently, no statistical differences were identified among KRAS^G12C^ and KRAS^non−G12C^ cases (Supplementary Figure S4A). Therapeutic responses to first-line therapy according to comutation are described in Supplementary Table S2. No concurrent mutations were significantly associated with ORR; however, compared to wild-type cases, tumors with GNAS (66.6% vs. 38.5%, OR 3.20, 95% CI 0.52 – 19.66. *p* = 0.209) and HER2 (66.7% vs. 38.5%, OR 3.20, 95% CI 0.52–19.66. *p* = 0.209) alterations demonstrated a tendency towards higher overall response rates. Conversely, STK11 (25.0% vs. 45.9%; OR 0.39, 95% CI 0.07–2.20, *p* = 0.288) and PI3KCA mutations (20.0% vs. 45.0%; OR 0.31, 95% CI 0.03–2.98, *p* = 0.308) exhibited lower ORR (Supplementary Figure S6). G12D subtype was the only factor independently associated with ORR in the entire cohort (OR 4.66, CI 95% 1.23–17.60, *p* = 0.023) (Supplementary Table S2).

At data cutoff, 3 (6.0%) patients remained without progression, and 11 (22.0%) were alive. Median duration of follow-up was 10.97 months (range 4.87–30.99). Median PFS (mPFS) was 6.01 months (95% CI 3.91– 7.36) in the entire cohort (Supplementary Figure S5A). No statistical differences in mPFS were observed according to KRAS mutation subtype; however, trend to higher among KRAS^G12D^ patients, compared to KRAS^non−G12D^ cases (8.28 vs. 4.34 months, HR 0.63, 95% CI 0.34–1.18, *p* = 0.100) (Fig. 3B). Meanwhile, no differences in mPFS were observed between KRAS^G12C^ and KRAS^non−G12C^ cases (Supplementary Figure S4B). Univariate analyses of factors associated with PFS *(*Table 2*)* were ECOG PS ≥ 2 (3.68 vs. 6.90 months; HR 2.48, 95% CI 1.22 – 5.06, p = 0.012) and clinical stage IV (4.34 vs. 10.38 months; HR 2.05, 95% CI 1.01–4.14, *p* = 0.045). According to co-occurring genomic status, mPFS was numerically shorter in patients with STK11 (4.34 vs. 6.47 months; HR 1.30, 95% CI 0.62–2.73, *p* = 0.483) and MET concurrent alterations (4.67 vs. 6.60 months; HR 2.12, 95% CI 0.83–5.49, *p* = 0.118). In multivariate analysis, only KRAS^G12D^ mutation (HR 0.36, 95% CI 0.16–0.80; *p* = 0.012) remained independently associated with prolonged PFS (Table 2). All patients’ median OS was 11.66 months (95% CI 7.36–25.33) (Supplementary Figure S5B); individuals with KRAS^G12D^ mutation showed significantly longer mOS (26.09 vs. 8.41, HR 0.46, 95% CI 0.23–0.95*, p* = 0.036) compared to KRAS^non−G12D^ cases (Fig. 3C). After adjusting for potential confounders, KRAS^G12D^ mutation (HR 0.24, 95% CI 0.08–0.70;* p* = 0.009) and ECOG PS ≥ 2 (HR 3.58; 95% CI 1.25–10.29, *p* = 0.018) were independently associated with OS (Table 3).

**Table 2:** Bivariate and multivariate analysis of progression-free survival

Characteristics					Bivariate analysis			Multivariate analysis		
	Events, n	mPFS (months)	95% CI	*P* value	HR	95% CI	*P* value	HR	95% CI	*P* value
Overall	48/50	6.01	3.91–7.36							
**Sex**										
Female	31/32	6.70	3.91–10.38		0.68	0.37–1.25				
Male	17/18	4.33	2.17–6.89	0.210*	1.47	0.80–2.69	0.214^**^			
**Age**										
≥ 65 years	23/24	7.06	2.33–6.70		0.75	0.43–1.34				
< 65 years	25/26	4.34	3.52–10.38	0.332*	1.32	0.75–2.34	0.335			
**ECOG PS **										
≥ 2	11/11	3.68	1.38–5.52		2.48	1.22–5.06		1.99	0.78–5.10	0.148
0–1	37/39	6.90	4.67–10.38	0.009*	0.40	0.20–0.82	**0.012**			
**Smoking status**										
Current/former smoker	28/30	6.60	2.33–7.36		0.74	0.42–1.33				
Never-smoker	20/20	5.52	2.96–10.97	0.313*	1.34	0.75–2.41	0.316			
**Wood-smoke exposure**										
Positive	13/13	5.52	3.68–10.38		1.31	0.69–2.50				
Negative	35/37	6.47	2.04–7.06	0.413*	0.76	0.40–1.46	0.415			
**Adenocarcinoma classification**										
LEP predominant	10/10	8.28	4.66–23.23		0.62	0.30–1.27	0.191			
PAP/ACN predominant	13/17	5.95	2.17–9.69		1.75	0.91–3.37	0.093			
SOL/MCP predominant	13/14	3.68	1.08–11.99	0.202*	0.89	0.45–1.78	0.753			
**Clinical stage**										
Stage IIIB	10/12	10.38	4.66–23.23		0.49	0.24–0.98				
Stage IV	38/38	4.34	2.76–6.90	0.040*	2.05	1.01–4.14	**0.045**	1.71	0.65–4.66	0.277
**Brain metastasis at diagnosis**										
Present	8/8	6.47	1.38–9.65		1.17	0.54–2.52				
Absent	40/42	5.95	3.67–8.28	0.687*	0.85	0.40–1.84	0.688			
**PD-L1 TPS expression**										
TPS ≥ 1%	17/18	9.66	3.91–14.62		0.60	0.29–1.25				
TPS < 1%	15/15	4.27	2.04–5.95	0.167*	1.66	0.80–3.41	0.172			
**PD-L1 TPS expression **										
TPS ≥ 50%	5/6	9.66	2.27–NR		0.31	0.09–1.06		0.32	0.10–1.00	**0.050**
TPS < 50%	27/27	5.22	2.76–7.36	0.083*	3.26	0.94–11.24	0.062			
**Tumor mutation burden**										
>10 mts/Mb	5/5	17.54	2.27–11.99		0.80	0.27–2.33				
< 10 mts/Mb	13/14	3.68	1.81—NR	0.676*	1.26	0.43–3.68	0.667			
**KRAS**^**G12C**^** subtype**										
KRAS^G12C^	15/16	4.67	2.04–7.06		1.20	0.64–2.23				
KRAS^non–G12C^	33/34	6.70	3.68–9.69	0.566*	0.83	0.45–1.55	0.567			
**KRAS**^**G12D**^** subtype**										
KRAS^G12D^	16/16	8.28	5.95–11.99		0.63	0.34–1.18		0.36	0.16–0.80	**0.012**
KRAS^non–G12D^	32/34	4.34	2.27–6.60	0.183*	1.57	0.84–2.91	0.100			
**TP53 mutation status**										
TP53^mt^	24/26	6.60	2.26–10.97		0.76	0.42–1.36				
TP53^wt^	24/24	5.22	2.96–7.36	0.259*	1.32	0.74–2.37	0.350			
**STK11 mutation status**										
STK11^mt^	9/9	4.34	1.08–13.27		1.30	0.62–2.73				
STK11^wt^	39/41	6.47	3.91–7.35	0.481*	0.77	0.37 1.61	0.483			
**GNAS mutation status**										
GNAS^mt^	6/6	5.95	4.27–NR		0.70	0.30–1.68				
GNAS^wt^	42/44	6.01	2.96–7.36	0.336*	1.41	0.59–3.36	0.434			
**HER2 mutations status**										
HER2^mt^	6/6	9.66	6.47–NR		0.76	0.42–1.36				
HER2^wt^	43/44	5.22	2.96–7.06	0.116*	1.98	0.78–5.07	0.350			
**CDKN2A alteration status**										
CDKN2A^del^	5/5	2.30	0.85–NR		0.87	0.31–2.44				
CDKN2A^wt^	43/45	6.47	4.27–7.36	0.955*	1.15	0.41–3.24	0.784			
**MET alteration status**										
MET^mt^	5/5	4.67	0.29–NR		2.12	0.83–5.49				
MET^wt^	43/45	6.60	3.90–8.28	0.109*	0.47	0.18–1.21	0.118			
**RB1 mutation status**										
RB1^mt^	5/5	6.90	3.52–NR		0.84	0.33–2.17				
RB1^wt^	43/45	5.95	3.68–8.28	0.620*	1.19	0.46–3.05	0.723			
**PI3KCA mutation status**										
PI3KCA^mt^	5/5	6.01	1.81–NR		0.81	0.32–2.08				
PI3KCA^wt^	43/45	6.90	3.68–7.36	0.564*	1.24	0.48–3.17	0.660			

*mPFS* median progression-free survival, *CI* confidence interval, *HR* hazard ratio. *ECOG PS* Eastern cooperative oncology group Performance Status, *LEP* lepidic, *CAN* acinar, *PAP* papillary, *MCP* micropapillary, *SOL* solid, *PD-L1 TPS* programmed death ligand 1 tumor proportion score, *Mts* mutations, *Mb* megabase, *KRAS* Kirsten rat sarcoma viral oncogene homolog, *G12C* missense substitution of glycine for cysteine*, G12D* missense substitution of glycine for aspartate, *TP53* tumor protein p53, *STK11* Serine/Threonine Kinase 11, *GNAS* guanine nucleotide binding protein, alpha stimulating. *HER2* human epidermal growth factor receptor 2, *CDKN2A* Cyclin-Dependent Kinase Inhibitor *2A. MET* mesenchymal epithelial transition, *RB1* Retinoblastoma 1, *PI3KCA* phosphatidylinositol-4,5-bisphosphate 3-kinase catalytic subunit alpha. Comparisons were performed using *Log-rank test. Statistically significant p values were determined as* p* ≤ 0.05 and shown as bold values in tables

**Table 3:** Bivariate and multivariate analysis of overall survival according to diverse clinical characteristics

Characteristics					Bivariate analysis			Multivariate analysis		
	Events, n	mOS (months)	95% CI	*p*-value	HR	95% CI	*p*–value	HR	95% CI	*p* value
Overall	39/50	11.66	7.36–25.33							
**Sex**										
Female	24/32	18.07	7.26–39.66		0.56	0.28–1.10				
Male	15/18	8.41	4.34–23.23	0.087*	1.80	0.91–3.56	0.092	2.11	0.72–6.19	0.174
**Age**										
≥ 65 years	18/24	12.65	7.36–39.66		0.92	0.49–1.76				
< 65 years	21/26	10.12	5.22–25.33	0.811*	1.08	0.57–2.05	0.811			
**ECOG PS **										
≥ 2	11/11	7.26	1.64–11.66		2.91	1.38–6.12		3.58	1.25–10.29	**0.018**
0–1	28/39	21.65	8.41–35.22	0.003*	0.34	0.16–0.72	**0.005**			
**Smoking status**										
Current/former smoker	24/30	11.03	5.39–25.33		0.93	0.48–1.79				
Never-smoker	15/20	11.66	4.67–39.66	0.830*	1.07	0.56–2.06	0.831			
**Wood-smoke exposure**										
Positive	12/13	7.49	2.03 -23.23		1.77	0.89–3.54				
Negative	27/37	12.65	7.36–30.65	0.099*	0.56	0.28–1.12	0.104			
**Adenocarcinoma classification**										
LEP predominant	9/10	9.07	4.67–30.65		1.16	0.53–2.55	0.707			
PAP/ACN predominant	12/17	23.23	7.26–46.88		0.83	0.40–1.70	0.606			
SOL/MCP predominant	11/14	8.41	1.08–47.70	0.867*	1.08	0.51–2.28	0.840			
**Clinical stage**										
Stage IIIB	7/12	28.48	6.47–NR		0.39	0.17–0.89				
Stage IV	32/38	8.41	5.22–21.65	0.021*	2.56	1.12–5.84	**0.026**	3.37	0.81–14.08	0.096
**Brain metastasis at diagnosis**										
Present	6/8	7.26	1.64–NR		0.94	0.40–2.27				
Absent	33/42	11.66	7.36–25.33	0.904*	1.05	0.44–2.53	0.905			
**PD-L1 TPS expression**										
TPS ≥ 1%	11/18	25.33	9.08–32.22		0.71	0.31–1.64				
TPS < 1%	12/15	7.36	2.76–NR	0.417*	1.41	0.61–3.25	0.420			
**PD-L1 TPS expression**										
TPS ≥ 50%	2/6	NR	25.33–NR		0.23	0.05–1.07		0.23	0.05–1.10	0.066
TPS < 50%	21/27	10.12	5.22–26.09	0.020*	4.32	0.93–20.01	0.061			
**Tumor mutation burden**										
>10 mt/Mb	4/5	28.48	5.39–NR		0.70	0.20–2.41				
< 10 mt/Mb	8/14	12.65	2.76–NR	0.571*	1.43	0.41–4.92	0.593			
**KRAS**^G12C^** subtype**										
KRAS^G12C^	14/16	5.22	2.37–23.23		1.80	0.91–3.56				
KRAS^non−G12C^	25/34	21.65	7.49–30.65	0.086*	0.55	0.28–1.10	0.091			
**KRAS**^G12D^** subtype**										
KRAS^G12D^	11/16	26.09	8.28–NR		0.46	0.23–0.95		0.24	0.08–0.70	**0.009**
KRAS^non–G12D^	28/34	8.41	4.34–23.06	0.032*	2.16	1.05–4.44	**0.036**			
**TP53 mutation status**										
TP53^mt^	21/26	23.23	6.47–30.65		0.69	0.36–1.33				
TP53^wt^	18/24	8.28	4.67–12.65	0.267*	1.44	0.75–2.78	0.270			
**STK11 mutation status**										
STK11^mt^	8/9	5.39	1.08–28.48		1.66	0.76–3.66				
STK11^wt^	31/41	12.65	7.49–26.09	0.199*	0.60	0.23–1.32	0.205			
**GNAS mutation status**										
GNAS^mt^	6/6	21.65	7.26–NR		0.87	0.36–2.10				
GNAS^wt^	33/44	11.04	5.38–23.23	0.760*	1.15	0.48–2.75	0.761			
**HER2 mutation status**										
HER2^mt^	4/6	11.04	6.47–NR		0.50	0.17–1.42				
HER2^wt^	35/44	11.66	5.39–23.23	0.183*	2.01	0.70–5.80	0.192			
**CDKN2A alteration status**										
CDKN2A^del^	4/5	12.65	0.86–NR		1.02	0.36–2.88				
CDKN2A^wt^	35/45	11.04	7.26–26.09	0.976*	0.98	0.35–2.79	0.977			
**MET alteration status**										
MET^mt^	5/5	4.67	8.28–26.09		1.64	0.62–4.38				
MET^wt^	34/45	12.65	0.30–NR	0.316*	0.61	0.23–1.62	0.321			
**RB1 mutation status**										
RB1^mt^	5/5	11.04	8.41–NR		0.76	0.27–2.15				
RB1^wt^	45/45	25.33	6.47–23.23	0.606*	1.31	0.46–3.71	0.608			
**PI3KCA mutation status**										
PI3KCA^mt^	5/5	12.65	7.49–NR		1.09	0.42–2.82				
PI3KCA^wt^	45/45	11.66	6.47–26.09	0.852*	0.91	0.35–2.35	0.853			

*mOS* median progression-free survival, *CI* confidence interval, *HR* hazard ratio, *ECOG PS* Eastern Cooperative Oncology Group Performance Status, *LEP* lepidic, *CAN* acinar, *PAP* papillary, *MCP* micropapillary, SOL solid, *PD-L1 TPS* programmed death ligand 1 tumor proportion score, *IO* immunotherapy, *KRAS* Kirsten rat sarcoma viral oncogene homolo, *G12C* missense substitution of glycine for cysteine, *G12D* missense substitution of glycine for aspartate, *TP53* tumor protein p53, *STK11* Serine/Threonine Kinase 11, *GNAS* guanine nucleotide binding protein, alpha stimulating. *HER2* human epidermal growth factor receptor 2. *CDKN2A* Cyclin-dependent kinase inhibitor *2A MET* mesenchymal epithelial transition. *RB1* Retinoblastoma 1, *PI3KCA* phosphatidylinositol-4,5-bisphosphate 3-kinase catalytic subunit alpha. Comparisons were performed using *Log-rank test. Statistically significant p values were determined as* p* ≤ 0.05 and shown as bold values in tables

### Impact of KRAS mutational subtype and concurrent mutations on immunotherapy efficacy

Since specific KRAS mutational subtypes and concurrent alterations may exert different effects on response and survival to immune checkpoint inhibitors (ICI), an exploratory efficacy analysis centered on subtype status and the two most prevalent alterations (TP53, STK11/KEAP1) in the cohort was performed. Objective response rate (50.0% vs. 39.4%, *p* = 0.524) and DCR (75.0% vs. 51.5%, *p* = 0.158) were numerically superior in patients receiving first-line PD-L1 blockade-based treatment compared to those receiving chemotherapy-based regimens. Overall, ORR (50.0% vs. 50.0%, *p* = 0.999) and DCR (75.0% vs. 75.0%, *p* = 0.999) were similar across KRAS^G12D^ and KRAS^non−G12D^ individuals (Fig. 3D)**.** Comparable response rates were observed in KRAS^G12C^ and KRAS^non−G12C^ cases (Supplementary Figure S4D). According to comutation status, a numerically lower ORR was described in cases with KRAS/TP53 (28.6% vs. 80.0%, *p* = 0.079) (Fig. 3G), as well as absence of response in KRAS/STK11 group (0.0% vs. 60.0%, *p* = 0.121) **(**Fig. 3J). Median PFS (10.38 vs. 5.95 months, HR 0.49, 95% CI 0.25–0.99, *p* = 0.047) and mOS (20.48 vs. 7.49 months, HR 0.43, 95% CI 0.21–0.89, *p* = 0.024) were significantly improved among individuals treated with immunotherapy. Among KRAS subtypes, not differences in mPFS after ICI were observed in KRAS^G12D^ group (9.69 vs. 7.35 months, *p* = 0.078) (Fig. 3E), while KRAS^G12C^ subgroup (Supplementary Figure S4E) showed better response. Differently, immunotherapy benefit in OS was consistent across KRAS^G12D^ individuals (NR vs. 11.66. *p* = 0.010) (Fig. 3F) and KRAS^G12C^ (Supplementary Figure S4F). Regarding comutations, KRAS/TP53 group showed a non-significant trend to longer mPFS (9.66 vs. 11.99 months, *p* = 0.078) (Fig. 3H) and a significantly better mOS (30.65 vs. 21.65 months,* p* = 0.030) (Fig. 3I) after immunotherapy, compared with wild-type TP53 group. Differently, KRAS/STK11 comutation harbored a trend to worse mPFS (4.33 vs. 11.99 months, HR 3.11, 95% CI 0.56–17.21, *p* = 0.251) (Fig. 3K) and as well as shorter mOS (21.65 vs. 30.65 months, HR 2.65, 95% CI 0.60–10.86, *p* = 0.049) (Fig. 3L).

## Discussion

This study provides valuable outcome information from a real-world cohort of Latin America patients with NSCLC harboring KRAS mutations and emphasizes the prognostic impact of diverse molecular profiles in this genomically-defined subset of lung cancer. Prevalence of KRAS mutations in our cohort significantly differs from studies conducted in Caucasian patients [10, 11], but aligns with that reported in Asian [12] and Latin American populations [2, 3]. This may be explained by a low tobacco smoke exposure; since we identified a higher proportion of never-smokers (40%) than in Caucasian populations (6.4–7.1%) [13], along with lower consumed pack per years (median 9.6) reported by smoker patients than previous studies (median 30.0) [5]. We found a higher proportion of KRAS^G12D^ cases compared with other cohorts [14], which agrees with available evidence not associating KRAS^G12D^ with smoking-related mutational signatures [13]. According to each mutational subtype, different carcinogenic patterns are activated, since KRAS^G12C^ triggers RalA/B signaling, while KRAS^G12D^ activates MEK and PI3K pathway [15].

KRAS^G12D^ exhibited a strong and independent association with favorable outcomes, conversely to previous evidence [5], likely explained by its infrequent concurrence with smoking-induced alterations, such as STK11 [16, 17], widely known to predict reduced survival rates and diminished clinical responses to systemic treatments [18]. In agreement, our observations suggested a deleterious prognostic effect of KRAS/STK11 comutation, also consistent with previous evidence in KRAS-mutated NSCLC [19]. Biological comprehension of this prognostic role has revealed that loss of STK11 impairs the activation of AMP-activated protein kinase (AMPK), consequently allowing activity of the mammalian target of rapamycin (mTOR) [20], ultimately inhibiting cell proliferation, cancer-associated metabolism, and differentiation towards metastatic phenotype [21]. These findings highlight the need for identification of agents capable of reactivating to improve patient outcomes. Regarding this, metformin restores AMPK-dependent signaling, leading to inhibition of tumor cell proliferation [22], but further prospective studies exploring its role in STK11-mutant NSCLC are warranted.

Differential survival outcomes among KRAS^G12D^ and KRAS^G12C^ cases may be driven by limited access to immunotherapy in our cohort. Consequently, deleterious responses and worse survival outcomes were noted among KRAS^G12C^ cases after treatment regimens without immunotherapy, which is consistent with previous findings [23]. Consequently, immunotherapy alone, or in combination, conferred a greater benefit in cases with KRAS^G12C^ mutation, as it is linked to a greater TMB in NSCLC, commonly associated to tobacco-related carcinogenesis [24], as well as more efficient tumor neoantigen presentation to T cells, higher infiltration of CD8^+^ T cells, and increased PD-L1 expression [5]. Meanwhile, KRAS^G12D^ subtype is associated with low PD-L1 expression and TMB, lack of pro-inflammatory IL-18 production, induction of CD3 + T cell apoptosis, and impairment of CD8 + T cell activation [25]. As well, the consistent benefit of immunotherapy in terms of overall survival along KRAS^G12D^ or KRAS^G12C^ groups may be derived from the impact of subsequent lines of treatment in KRAS^G12D^ cases and concomitant employment of chemotherapy in almost all patients undergoing ICI-based regimens. Nevertheless, insufficient statistical power avoided comparing first-line monotherapy with PD-L1 blocking and chemoimmunotherapy in this population.

Moreover, KRAS^G12D^-mutated NSCLC may harbor exceptional oncogenic biology and treatment response. Regarding the coalterations, we found a higher incidence of uncommon EGFR comutations (14%) in almost all KRAS^G12D^ cases, contrasting with available literature in Western individuals with KRAS mutations (1.3–4.0%) [14, 26]. As well, other comutations constituted predictive biomarkers of response to PD-L1 blockade. Specifically, STK11 was related to shorter PFS and OS, in line with previous reports [18, 27], but limited sample size prevented statistical significance. Biological reasoning underlining these findings describes a lack of PD-L1 expression and lower densities of infiltrating CD8^+^ T cells in STK11-altered tumors [18]. Consequently, STK11/LKB1 co-alteration is widely known as an independent predictor of unfavorable outcomes after PD-L1 blockade in lung adenocarcinoma [28]. Thereby, it has been theorized that a triple regimen comprised of chemotherapy plus PD-L1 and CTLA-4 blockade may improve clinical response of this hard-to-treat subgroup [29]. Differently, is consistent with available literature describing that TP53 comutations show a remarkable benefit of PD-L1 blockade, likely derived from a TP53-related increase in PD-L1 expression and a greater infiltration of CD8^+^ T-cells in lung adenocarcinomas [30].

Limitations of the present study need to be considered when interpreting these results. Firstly, limited sample size in our cohort may have reduced the statistical power to detect significant differences among subgroups harboring distinct co-occurring genomic alterations. Secondly, information regarding PD-L1 expression was unavailable for all patients; therefore, we were not able correlate TMB and PD-L1 expression with distinct biological subgroups in the cohort. Thirdly, a low availability of immunotherapy-based regimens conditioned that only a minority of patients were treated with this therapeutic modality, hindering performance of a multivariate analysis evaluating factors associated with ICI-related clinical outcomes.

## Conclusions

To our knowledge, this study represents the first effort to comprehensively characterize the molecular heterogeneity of KRAS-mutant NSCLC in Latin American patients. Our data reinforce the current view that KRAS-mutated NSCLC is not a single oncogene-driven disease and emphasizes the prognostic impact of diverse molecular profiles in this genomically defined subset of NSCLC. Further validation is warranted in larger multicenter Latin American cohorts to confirm our findings.

### Supplementary Information

Below is the link to the electronic supplementary material.

Supplementary file1 (PDF 1193 KB)

Supplementary file2 (PDF 438 KB)

## Data Availability

Not applicable.
