# Supplementary material for: Impact of KRASG12D subtype and concurrent pathogenic mutations on advanced non-small cell lung cancer outcomes
Source: Clin Transl Oncol. 2023 Jul 25;26(4):836–50. doi: 10.1007/s12094-023-03279-2 (PMC10981588; doi:10.1007/s12094-023-03279-2)
Supplement: Supplementary file 1 — Supplementary file1 (PDF 1193 KB) [file 12094_2023_3279_MOESM1_ESM.pdf]

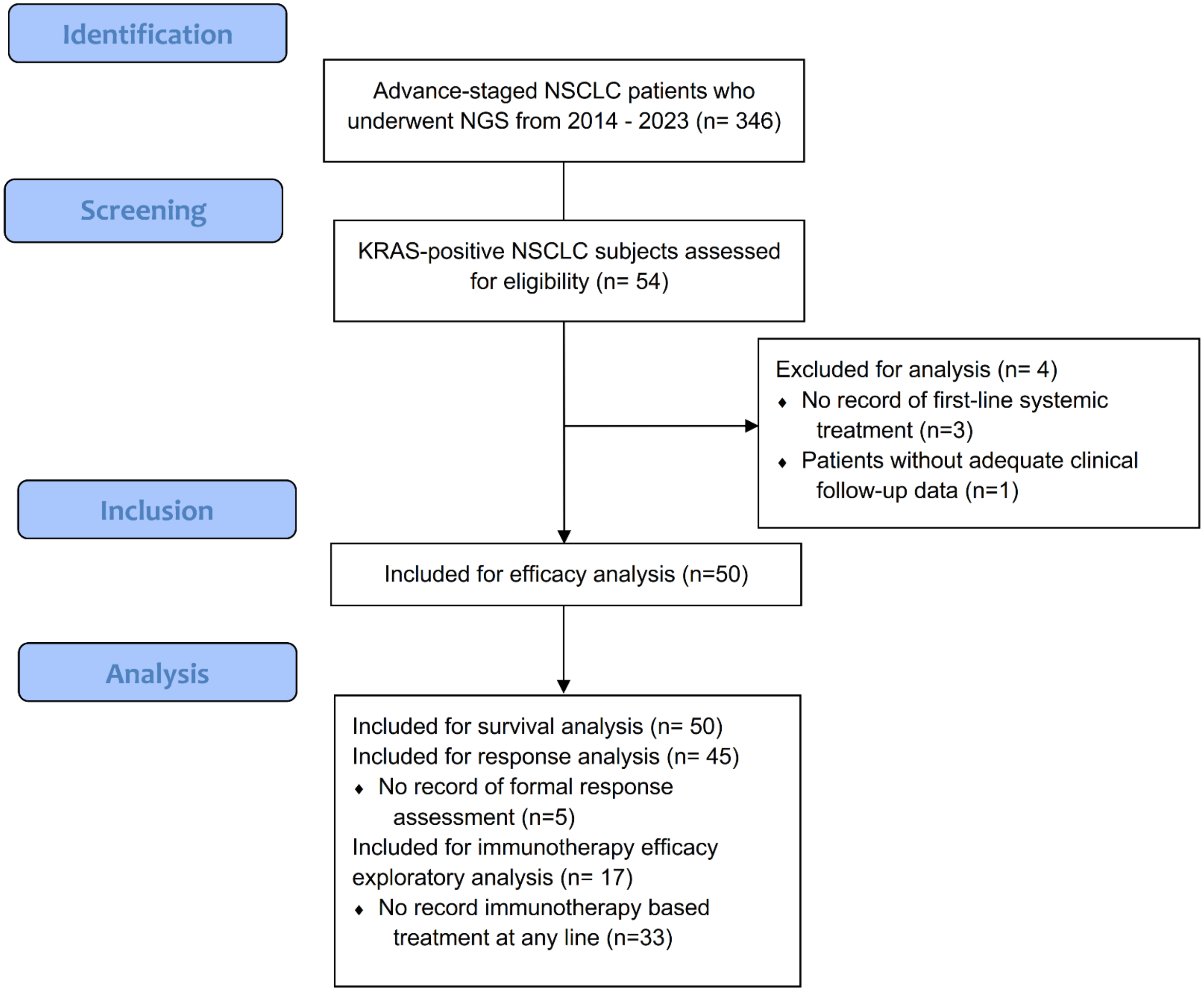

Supplementary Figure 1. Eligibility assessment of KRAS-mutated NSCLC individuals for efficacy analysis. NSCLC, Non-Small Cell Lung Cancer. NGS, Next-Generation Sequencing. KRAS, Kirsten rat sarcoma viral oncogene homolog.

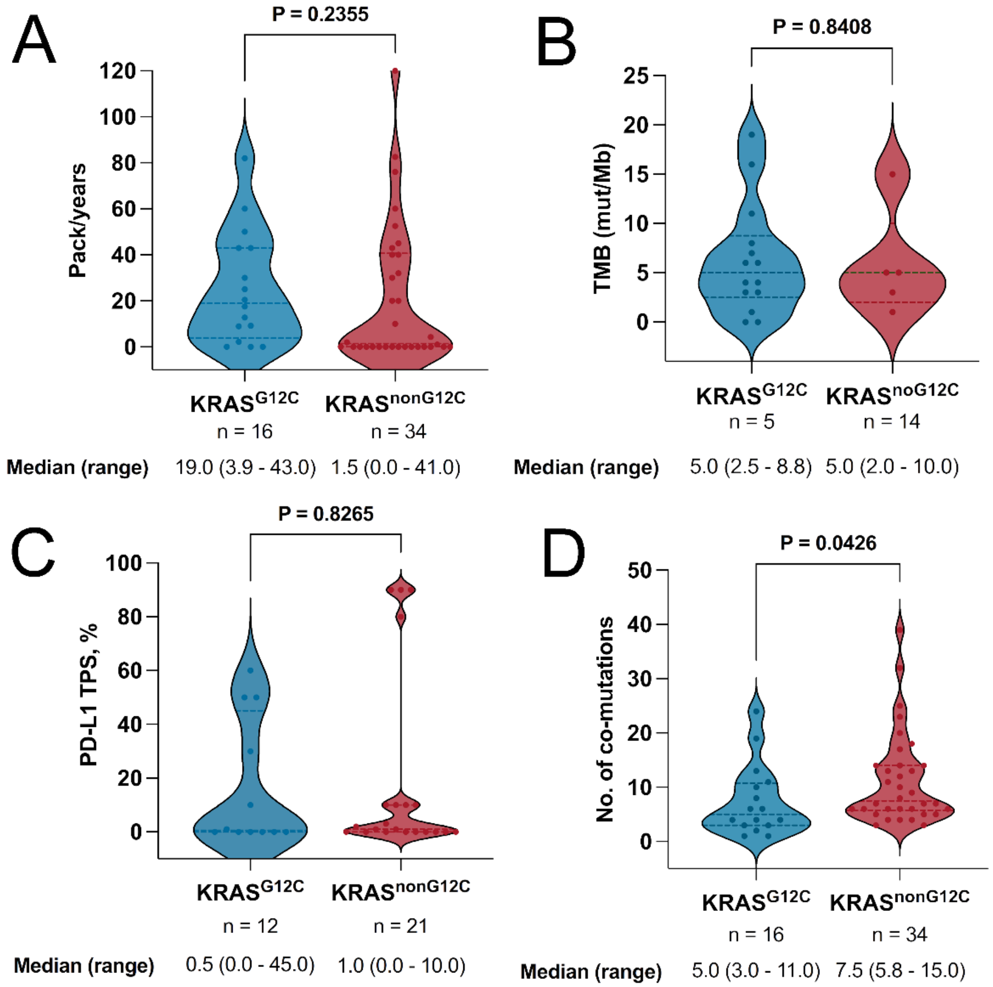

Supplementary Figure 2. Smoking history according to packs per year (A), tumor mutational burden (B), PD-L1 TPS (C), and number of co-mutations (D) in individuals with KRAS G12C or non-G12C mutations. KRAS, Kirsten rat sarcoma viral oncogene homolog. G12C, missense substitution of glycine for cysteine. Comparisons were made using Mann-Whitney test according to normal distribution determined by the Kolmogorov-Smirnov test. Significant *p* values were defined as less than 0.05.

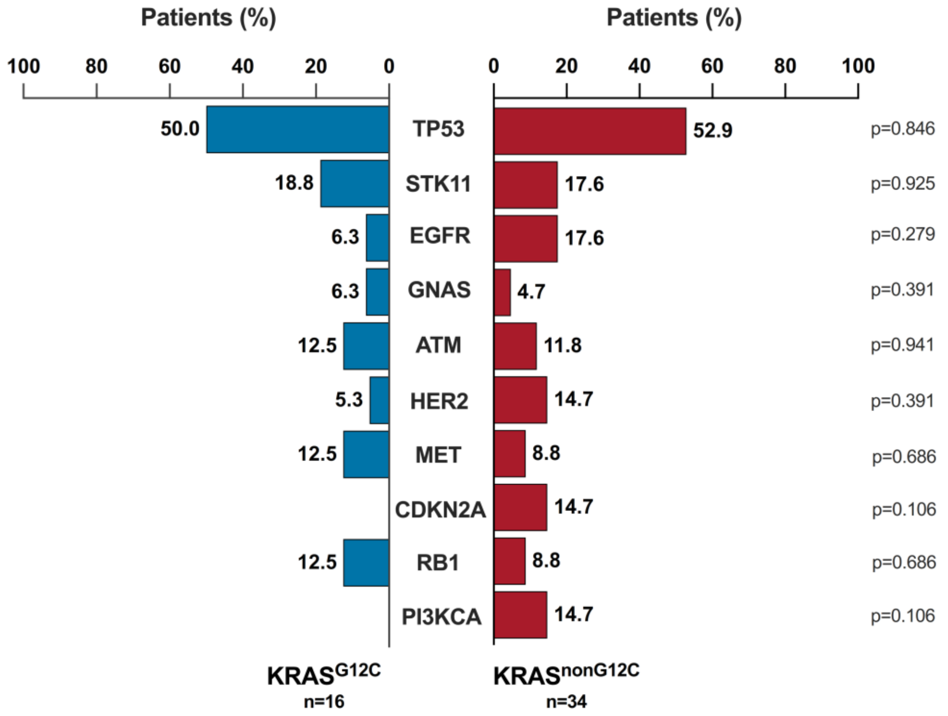

Supplementary Figure 3. Prevalence of co-mutations in individuals with G12C mutations. KRAS, Kirsten rat sarcoma viral oncogene homolog. G12C, missense substitution of glycine for cysteine. TP53, tumor protein p53. STK11, Serine/Threonine Kinase 11. EGFR, Epidermal Growth Factor Receptor. GNAS, guanine nucleotide-binding protein, alpha stimulating. ATM, Ataxia-Telangiectasia Mutated. HER2, human epidermal growth factor receptor 2. MET, mesenchymal-epithelial transition factor. CDKN2A, Cyclin-Dependent Kinase Inhibitor 2A. RB1, Retinoblastoma 1. PI3KCA, phosphatidylinositol-4,5-bisphosphate 3-kinase catalytic subunit alpha. Comparisons were performed by Pearson Chi-Square test. Significance was set at *p* values <0.05.

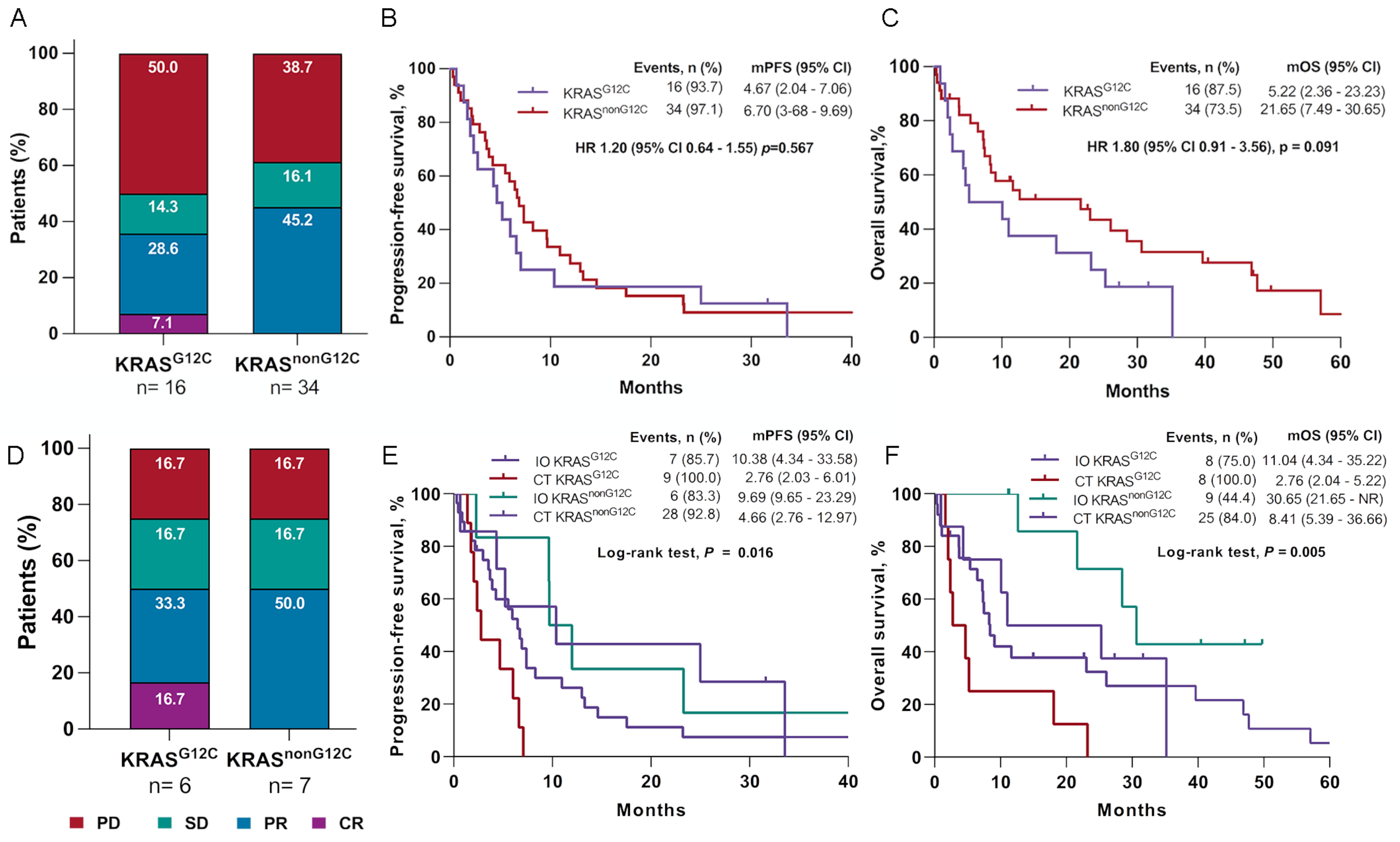

Supplementary Figure 4. A, therapeutic responses to all treatments in individuals with KRAS G12C or non-G12C mutations. **B**, progression-free survival of individuals with KRAS G12C or non-G12C mutations after all treatments. **C**, overall survival of patients having KRAS G12C or non-G12C mutations after all treatments. **D**, therapeutic responses to immunotherapy according to KRAS G12C mutation. **E**, progression-free survival of individuals having G12C or non-G12C KRAS mutations undergoing immunotherapy or chemotherapy. **F**, overall survival of individuals having G12C or non-G12C KRAS mutations undergoing immunotherapy or chemotherapy. HR, hazard ratio. IO, immunotherapy. ICI, immune checkpoint inhibitors. CT, chemotherapy. PFS, progression-free survival. OS, overall survival. KRAS, Kirsten rat sarcoma viral oncogene homolog. G12C, missense substitution of glycine for cysteine. PFS was calculated from diagnosis to progression to first-line treatment. OS was determined by the period between diagnosis and death for any cause. Log-rank test was performed to determine statistical differences between Kaplan-Meyer curves as always as *p* values were less than 0.05.

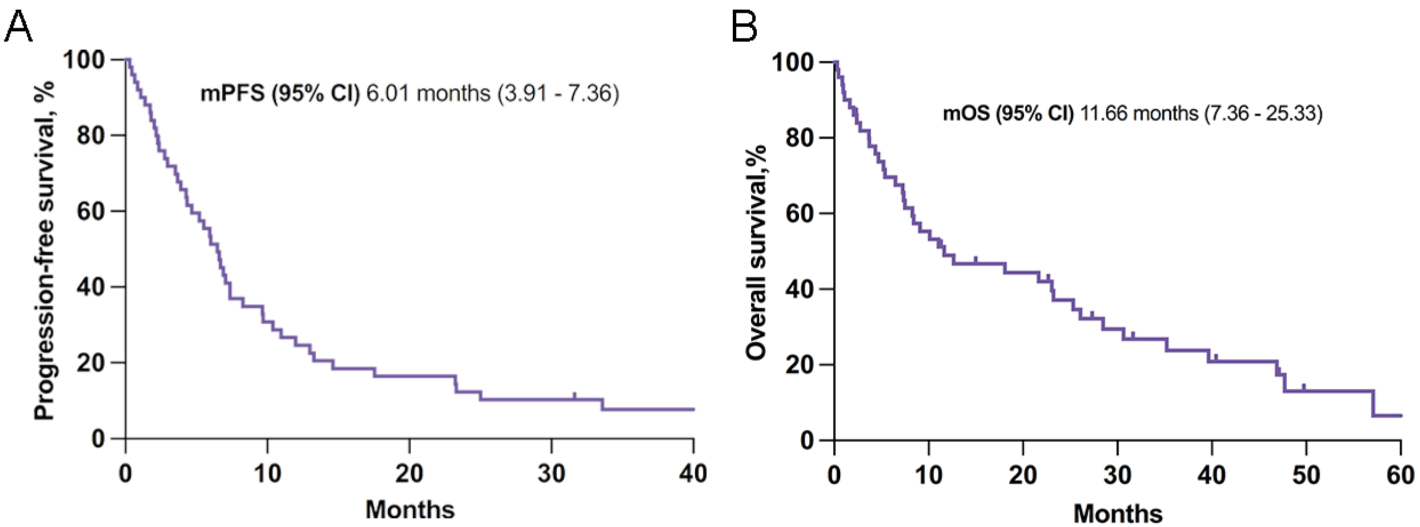

Supplementary Figure 5. A, progression-free survival of individuals with KRAS mutations. **B,** overall survival in patients with KRAS mutations. mPFS, median progression-free survival. mOS median overall survival. CI, confidence interval. PFS was calculated from diagnosis to progression to first-line treatment. OS was determined by the period between diagnosis and death for any cause. Log-rank test was performed to determine statistical differences between Kaplan-Meyer curves as always as *p* values were less than 0.05.

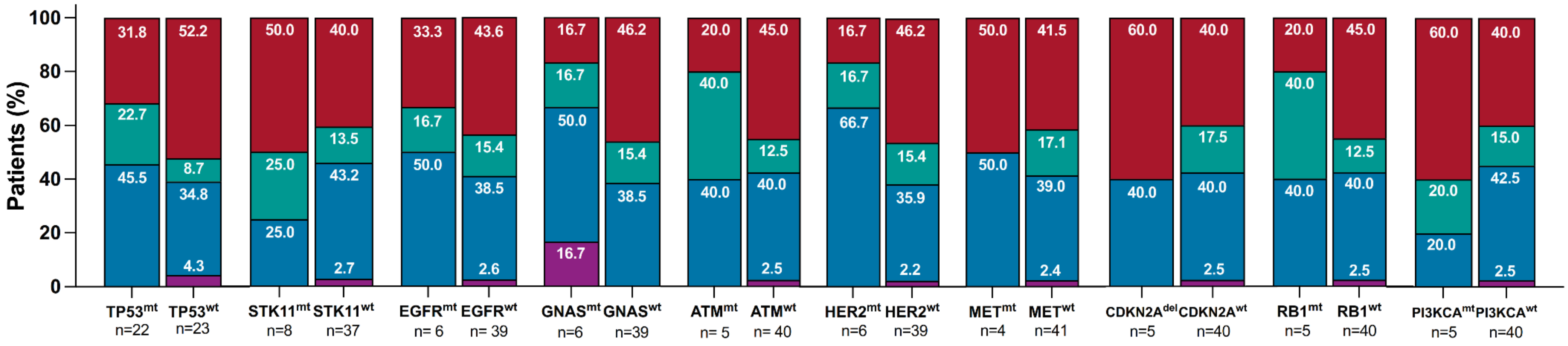

Supplementary Figure 6. Therapeutic responses to all treatments in individuals with diverse comutations. TP53, tumor protein p53. STK11, Serine/Threonine Kinase 11. EGFR, epidermal growth factor receptor gene. GNAS, guanine nucleotide-binding protein, alpha stimulating complex locus. ATM, ataxia telangiectasia mutated. HER2, human epidermal growth factor receptor 2. CDKN2A, Cyclin-Dependent Kinase Inhibitor 2A. RB1, Retinoblastoma 1. PI3KCA, phosphatidylinositol-4,5-bisphosphate 3-kinase catalytic subunit alpha.
