# Supplementary material for: Impact of KRASG12D subtype and concurrent pathogenic mutations on advanced non-small cell lung cancer outcomes
Source: Clin Transl Oncol. 2023 Jul 25;26(4):836–50. doi: 10.1007/s12094-023-03279-2 (PMC10981588; doi:10.1007/s12094-023-03279-2)
Supplement: Supplementary file 2 — Supplementary file2 (PDF 438 KB) [file 12094_2023_3279_MOESM2_ESM.pdf]

		Total	KRAS^{G12C}	KRAS^{noG12C}	*P value*
		N= 50 (100.0)	n= 16 (32.0)	n= 34 (68.0)	
**Age, mean (±SD)**		62.8 (11.6)	62.6 (8.5)	62.9 (12.9)	0.461ǂ
**Sex, n (%)**	Male	18 (36.0)	6 (37.5)	12 (35.3)	
	Female	32 (64.0)	10 (62.5)	22 (64.7)	0.880†
**ECOG PS, n (%)**	0-1	39 (78.0)	13 (81.3)	26 (76.5)	
	≥2	11 (22.0)	3.0 (18.8)	8.0 (23.5)	0.704†
**Smoking status, n (%)**	Current/former	30 (60.0)	13 (81.3)	17 (50.0)	
	Never	20 (40.0)	3 (18.7)	17 (50.0)	**0.035**
**Pack-years, median (range)**		9.6 (0.0 – 43.0)	19.0 (3.9 – 43.0)	1.5 (0.0 – 41.0)	0.235§
**WSE, n (%)**	Positive	13 (26.0)	5 (31.2)	8 (23.5)	
	Negative	37 (74.0)	11 (68.8)	26 (76.5)	0.562†
**Hours/years, median (range)**		0.0 (0.0 – 4.37)	0.0 (0.0 – 35.0)	0.0 (0.0 – 0.6)	0.425§
**Histology, n (%)**	Adenocarcinoma	49 (98.0)	16 (100.0)	33 (97.1)	1.00
**Adenocarcinoma classification, n (%) (n= 45)**	LEP predominant	10 (22.2)	2 (15.3)	8 (25.0)	
	ACN predominant	13 (28.9)	5 (38.5)	8 (25.0)	
	PAP predominant	4 (8.9)	1 (7.7)	3 (9.4)	
	MCP predominant	1 (2.2)	0 (0.0)	1 (3.12)	
	SOL predominant	17 (37.8)	5 (38.5)	12 (37.5)	0.884‡
**Clinical stage, n (%)**	Stage IIIB-C	12 (24.0)	4 (25.0)	8 (23.5)	
	Stage IVA-IVB	38 (76.0)	12 (75.0)	26 (76.5)	0.910†
**PD-L1 expression, n (%) (n = 33)**	TPS <1%	15 (45.5)	6 (50.0)	9 (43.9)	
	TPS ≥1%	18 (54.5)	6 (50.0)	12 (57.1)	0.692†
**PD-L1 expression, n (%) (n = 33)**	TPS <50%	27 (81.8)	9 (75.0)	18 (85.7)	
	TPS ≥50%	6 (18.2)	3 (25.0)	3 (14.3)	0.443‡
**PD-L1 TPS, median (range)**		1.0 (0.0 – 20.0)	0.50 (0.0 – 45.0)	1.0 (0.0 – 10.0)	0.826§
	Not assessed	17	4	13	
**TMB, median (range) (n = 19)**		5.0 (3.0 – 8.0)	5.0 (2.5 – 8.8)	5.0 (2.0 – 10.0)	0.840§
**TMB, n (%) (n = 19)**	<10mt/MB	14 (73.7)	3 (60.0)	11 (78.6)	
	≥10mt/MB	5 (26.3)	2 (40.0)	3 (21.4)	0.570‡
	Not assessed	31	11	20	
**No. co-occurring mutations, median (range)**		7.0 (4.0 -13.3)	5.0 (3.0 – 11.0)	7.5 (5.8 – 15.0)	**0.0426**§
**Metastatic sites, n (%) (n = 38)**	Lymph nodes	10 (20.0)	0.0 (0.0)	10 (29.4)	**0.015**†
	Contralateral lung	20 (40.0)	6 (37.5)	14 (41.2)	0.804†
	Pleura	7 (14.0)	2 (12.5)	5 (14.7)	0.834†
	Bone	16 (32.0)	6 (37.5)	10 (29.4)	0.567
	CNS	8 (16.0)	3 (18.8)	5 (14.7)	0.716

Supplementary Table 1. Clinical characteristics of patients with KRAS G12C mutation. KRAS, KRAS, Kirsten rat sarcoma viral oncogene homolog. G12C, missense substitution of glycine for cysteine. ECOG, Eastern Cooperative Oncology Group Performance Status. WSE, wood smoke exposure. LEP, lepidic. ACN, acinar. PAP, papillary. MCP, micropapillary. SOL, solid. TPS, tumor proportion score. PD-L1 TPS, programmed death ligand 1 tumor proportion score. TMB, tumor mutational burden. EGFR, Epidermal Growth Factor Receptor. TKI, tyrosine kinase inhibitor. CNS, central nervous system. Mts, mutations. MB, megabase. Comparisons were made using ƚ t-test or §Mann-Whitney test according to the normal distribution determined by the Kolmogorov-Smirnov test. Nominal variables were analyzed by †Pearson Chi-Square test, except where small size (n < 5) required the use of ‡Fisher's exact test. Significance was set at $p < 0.05$ (two-sided).

	Response rate					
	**ORR (%)**	**95% CI**	***P* value**	**OR**	**95 CI%**	***P***
**Overall**	42.0%	27.7 – 57.8				
**Sex**						
Female	50.0	0.36 – 0.68		2.40	0.66 – 8.63	0.180
Male	29.4	0.12 – 0.55	0.175	1.00	Ref.	
**Age**						
≥65 years	40.9	0.22 – 0.63		0.90	0.27 – 2.94	0.862
<65 years	43.5	0.24 – 0.64	0.862	1.00	Ref.	
**ECOG PS (≥2)**						
≥2	40.0	0.14-0.72		0.88	0.21 – 3.72	0.872
0 –1	42.9	0.27-0.60	0.872*	1.00	Ref.	
**Smoking status**						
Current or former	34.5	0.19-0.54		0.40	0.12 – 1.43	0.161
Non-smoker	56.2	0.31-0.79	0.157*	1.00	Ref.	
**WSE**						
Positive	66.6	0.31-0.90		3.54	0.76 – 16.57	0.109
Negative	36.1	0.22-0.53	0.097*	1.00	Ref.	
**Histological grade (High grade)**						
Low	25.0	0.05-0.66		0.42	0.07 – 2.45	0.342
Intermediate	47.3	0.26-0.70		1.80	0.50-6.46	0.367
High	38.4	0.16-0.67	0.188*	1.00	Ref.	
**Adenocarcinoma classification**						
LEP predominant	44.4	0.16-0.77		1.20	0.27 – 5.40	0.812
PAP/ACN predominant	41.1	0.20-0.66		1.01	0.28 – 3.66	0.987
SOL/MCP predominant	38.5	0.16-0.68	0.961*	1.00	Ref.	
**PDL1 expression status (positive vs. negative)**						
TPS >1%	43.8	0.21-0.69		1.03	0.24 – 4.41	0.961
TPS <1%	42.9	0.19-0.70	0.961*	1.00	Ref.	
**PDL1 expression status (high vs. low)**						
TPS ≥50%	40.0	0.07-0.85		0.85	0.12 – 6.00	0.869
TPS <50%	42.1	0.21-0.66	0.932*	1.00	Ref.	
**TMB ≥ 10 mt/MB (Present)**						
≥ 10 mt/MB	40.0	0.07-0.85		2.22	0.24 –20.17	0.478
< 10 mt/MB	23.1	0.07-0.56	0.583**	1.00	Ref.	
**KRAS subtype (G12C)**						
$KRAS^{G12C}$	35.7	0.15-0.64		0.67	0.18 – 2.48	0.554
$KRAS^{nonG12C}$	45.2	0.28-0.63	0.553*	1.00	Ref.	
**KRAS subtype (G12D)**						
$KRAS^{G12D}$	66.6	0.39-0.86		4.66	1.23 – 17.60	**0.023**
$KRAS^{nonG12D}$	30.0	0.16-0.49	0.019*	1.00	Ref.	
**KRAS concurrent mutation**						
$TP53^{mt}$	45.5	0.26-0.67		1.29	0.40 – 4.24	0.668

TP53^{wt}	39.1	0.21-0.61	0.668*			
STK11^{mt}	25.0	0.05-0.66		0.39	0.07 – 2.20	0.288
STK11^{wt}	45.9	0.30-0.63	0.277*			
EGFR^{mt}	50.0	0.14-0.86		1.46	0.26 – 8.05	0.680
EGFR^{wt}	41.0	0.26-0.57	0.679*			
GNAS^{mt}	66.6	0.23-0.93		3.20	0.52 – 19.66	0.209
GNAS^{wt}	38.5	0.24-0.55	0.193*			
ATM^{mt}	40.0	0.07-0.84		0.90	0.14 – 6.01	0.915
ATM^{wt}	42.5	0.27-0.59	0.915**			
HER2^{mt}	66.6	0.23-0.93		3.20	0.52 – 19.67	0.209
HER2^{wt}	38.5	0.24-0.55	0.377**			
CDKN2A^{del}	40.0	0.08-0.84		0.90	0.14 – 6.01	0.916
CDKN2A^{wt}	42.5	0.27-0.59	0.915**			
MET^{mt}	50.0	0.08-0.91		1.41	0.18 – 11.03	0.742
MET^{wt}	41.4	0.27-0.57	0.741**			
RB1^{mt}	40.0	0.08-0.84		0.90	0.14 – 6.01	0.916
RB1^{wt}	42.5	0.28-0.59	0.915**			
PI3KCA^{mt}	20.0	0.02-0.76		0.31	0.03 – 2.98	0.308
PI3KCA^{wt}	45.0	0.30-0.61	0.378**			

Supplementary Table 2. Objective response rate to all treatments according to diverse clinical characteristics. ORR, objective response rate. IO, immunotherapy. OR odds ratio. ECOG, Eastern Cooperative Oncology Group Performance Status. WSE, Wood smoke exposure. PD-L1 TPS, programmed death ligand 1 tumor proportion score. TMB, Tumor Mutational Burden. KRAS, Kirsten rat sarcoma viral oncogene homolog. G12C, missense substitution of glycine for cysteine. G12D, missense substitution of glycine for aspartate. TP53, tumor protein p53. STK11, Serine/Threonine Kinase 11. EGFR, epidermal growth factor receptor gene. GNAS, guanine nucleotide-binding protein, alpha stimulating complex locus. ATM, ataxia telangiectasia mutated. HER2, human epidermal growth factor receptor 2. CDKN2A, Cyclin-Dependent Kinase Inhibitor 2A. MET, mesenchymal-epithelial transition factor. RB1, Retinoblastoma 1. PI3KCA, phosphatidylinositol-4,5-bisphosphate 3-kinase catalytic subunit alpha. Comparisons were performed using *Chi quare test, **Fisher. Statistically significant *p* values were determined as $p \leq 0.05$.

Treatment regimen	Total	
	N = 50	
**First-line treatment, n (%)**	**50 (100.0)**	
**Anti-PD-(L)1 monotherapy, n (%)**	**2 (4.0)**	
Pembrolizumab, n (%)	2 (4.0)	
**Anti-PD-(L)1 monotherapy + platinum based-chemotherapy, n (%)**	**11 (22.0)**	
Carboplatin/Pemetrexed/ Pembrolizumab, n (%)	8 (16.0)	
Carboplatin/Pemetrexed/Durvalumab, n (%)	3 (6.0)	
**Platinum based-chemotherapy, n (%)**	**36 (72.0)**	
Carboplatin/Pemetrexed, n (%)	21 (42.0)	
Carboplatin/Gemcitabine, n (%)	1 (2.0)	
Carboplatin/Paclitaxel, n (%)	13 (26.0)	
Carboplatin/Paclitaxel/Bevacizumab, n (%)	1 (2.0)	
**Targeted therapy, n (%)**	**1 (2.0)**	
Erlotinib, n (%)	1 (2.0)	
**Second-line treatment, n (%)**	**24 (48.0)**	
**Anti-PD-(L)1 monotherapy****	**6 (12.0)**	
Pembrolizumab, n (%)	3 (6.0)	
Atezolizumab, n (%)	1 (2.0)	
Nivolumab, n (%)	2 (4.0)	
**Chemotherapy, n (%)**	**16 (32.0)**	
Carboplatin/Pemetrexed, n (%)	3 (6.0)	
Carboplatin/Paclitaxel, n (%)	1 (2.0)	
Carboplatin/Paclitaxel/Bevacizumab, n (%)	1 (2.0)	
Docetaxel, n (%)	6 (12.0)	
Gemcitabine, n (%)	3 (6.0)	
Pemetrexed, n (%)	2 (4.0)	
**Targeted therapy, n (%)**	**2 (4.0)**	
Afatinib, n (%)	1 (2.0)	
Sotorasib, n (%)	1 (2.0)	

Supplementary Table 3. Therapeutic regimens employed in the cohort. PD-L1, programmed death ligand 1. PD, progressive disease.
**Two patients continue anti-PD-(L)1 monotherapy after progressive disease.
